# The Mystery of Ehlers-Danlos Syndrome: An Autobiographical Case Report

**DOI:** 10.7759/cureus.21601

**Published:** 2022-01-25

**Authors:** Brandon E Tapasak, David J Malis

**Affiliations:** 1 College of Medicine, University of Central Florida, Orlando, USA; 2 Otolaryngology, My Family ENT, Melbourne, USA

**Keywords:** connective tissue, psychosocial, fatigue, gastroesophageal reflux disease, pneumothorax, ehlers-danlos syndrome, autobiographical case report

## Abstract

Ehlers-Danlos syndrome (EDS) most often presents with the classic symptoms of skin hyperelasticity, hypermobility of joints, atrophic scarring, and fragility of blood vessels. However, EDS can also have uncommon presentations which are much more insidious. This case report details the author’s lifelong experience living with EDS, which was diagnosed after many seemingly unrelated afflictions including fatigue, spontaneous pneumothorax, and gastroesophageal reflux disease. Studies indicate that these complications warrant investigation of the connective tissue disorder with further lifelong follow-up of disease progression. Extra care should be taken to differentiate the disorder from other heritable connective tissue disorders as well as consider the psychosocial issues these patients experience.

## Introduction

Ehlers-Danlos syndrome (EDS) affects collagen formation and function of every organ system, resulting in significant morbidity and mortality. Known common complications include arterial rupture, organ rupture, joint dislocation, chronic pain, and fatigue [1]. Lesser-known complications include gastroesophageal reflux disease (GERD) and spontaneous pneumothorax.

The most recent literature characterizes 14 different types of EDS, thirteen of which have an identified molecular cause [2]. Due to the diverse presentations of EDS, the syndrome can be difficult for the clinician to diagnose. The diagnostic challenge of EDS is further complicated by overlapping symptomatology with hypermobility spectrum disorders, Marfan syndrome (MFS), Loeys-Dietz syndrome (LDS), Cutis Laxa syndrome, polycystic kidney disease, osteogenesis imperfecta (OI), and chronic fatigue syndrome (CFS) [3]. Therefore, it is essential for clinicians to have good clinical suspicion and curiosity for adequate diagnosis.

This case report details the author’s experience living with EDS, including complications and impact on the quality of life consequent to delay in diagnosis. The goal of writing this autobiographical case report is to provide a resource regarding rare presentations of EDS, highlight a need for improved clinical diagnosis, and improve the understanding of the psychosocial issues these patients face.

## Case presentation

I was diagnosed with classical EDS (cEDS) in 2015 during my sophomore year of undergraduate school at 20 years of age. My progression through medical school and increased understanding of what the condition entails have made me more aware of the long-term consequences for myself as well as my future children. The following report details the complications I had never known were abnormal until doing my research, followed by the psychosocial ramifications for myself and other patients who experience more common presentations.

Spontaneous pneumothorax

In 2010, I was 15 years old and walking to lunch from Spanish class when I experienced excruciating chest pain and shortness of breath. This was the first spontaneous pneumothorax, diagnosed after a chest X-ray at our local hospital. Approximately 40% of my left lung had collapsed. I have always been a tall, thin male; the classic patient population that pneumothoraces affect. As such, this was my diagnosis. My pulmonologist indicated this was not likely to happen again. I returned to school after a week-long stay in the hospital.

Six months later, I was running with my friends when I experienced the same pain and dyspnea. This time I knew what had happened. However, this time the pneumothorax collapsed approximately 80% of my lung. For this ailment, we traveled to an acute pediatric care hospital. After a CT scan, our pulmonologist advised a partial lung resection to remove the blebs that could cause more pneumothoraces.

After undergoing a left lung wedge resection, our pulmonologist referred me to a genetic specialist for an MFS evaluation. Spontaneous pneumothorax is part of the “systemic score” used for clinical evaluation when suspecting MFS. This was the first time we considered an underlying etiology. However, we did not follow through with genetic testing due to the absence of aortic root dilation and ectopia lentis as well as cost limitations. Even if there was a genetic mutation present, I did not meet the criteria for diagnosis. Other than the tall, thin frame, I “didn’t look like a Marfan patient.” The classic presentations of wrist and thumb signs, chest deformities, scoliosis, and other systemic manifestations were not present. We did not consider other diagnoses, missing an opportunity for an earlier diagnosis. Since these two episodes, I have not experienced any subsequent spontaneous pneumothoraces.

Gastroesophageal reflux disease

I have experienced issues with eating my entire life. I would often stop in the middle of a meal to “wait for food to go down.” These symptoms felt like dry food taking longer than usual to pass, causing me to pause eating and swallowing for seconds to minutes until the food bolus moved down my esophagus. This pseudo-dysphagia prolonged meals and resulted in eating less. Additionally, I would feel burning chest pain and regurgitation, especially after meals and in the middle of the night when sleeping. My worst triggers were tomato sauce and coffee. With a background in healthcare, my mother recognized my symptoms as abnormal when I was 19 years old. A barium esophagram confirmed free reflux at the gastroesophageal junction. Our gastroenterologist suggested a proton pump inhibitor (PPI) regimen for eight weeks. Although my symptoms improved for the first two weeks, they returned over the following weeks. These refractory symptoms indicated further investigation. Manometry revealed a maximum lower esophageal sphincter pressure of 9 mmHg with normal relaxation on swallowing and preserved peristaltic waves. Twenty-four-hour ambulatory pH monitoring revealed a DeMeester score (DMS) of 110 (DMS >100 indicates severe GERD). The endoscopy with biopsy showed Barret’s esophagitis limited to the gastroesophageal junction. When lifestyle changes in addition to the medication did not help, we elected to pursue surgery. I underwent a laparoscopic magnetic sphincter augmentation which has made my symptoms more manageable. The surgical procedure reproduces the lower esophageal sphincter tone to permit food passage into the stomach but prevents acid from refluxing into the esophagus. While long-term data is still being collected, we chose the procedure because of the ease of reversal, unlike the Nissen fundoplication. A follow-up endoscopy when I was 21 years old showed regression of Barret’s epithelium. The repeat pH monitoring revealed a DMS of 15 (DMS <14.72 indicates no GERD and DMS of 14.72-50 indicates mild GERD). Although I have improved postoperatively, I start a PPI regimen to handle stress-related symptom exacerbations.

Fatigue

In high school, I was six feet six inches in height and 190 pounds in weight with a body mass index of 22. The tallest person in my class, people assumed I was a great basketball player and had the opportunity to play competitively. But this could not be further from the truth. When I played competitive basketball, I fell behind my teammates. My conditioning was consistently behind my peers no matter how much I trained. I was told I “wasn’t working hard enough” so often I believed it. I was so tired all the time. I returned home from practice and could not move. I dragged through classes the next day until going to basketball practice again that afternoon. My feet felt like rocks weighing me down. To me, this was normal until the geneticist who diagnosed me with EDS asked, “did you feel like you physically couldn’t keep up with your friends?” Although I experienced this all my life, the drawbacks were most apparent during high school. I have learned how to manage my symptoms with an exercise schedule, healthy eating habits, and goals to keep me focused. The fatigue is only present during physical activity and has not worsened over time. Admittedly, in medical school, I am not as active as I used to be. However, when running or exercising with friends I tire easier and am lightheaded before forcing myself to rest.

Cardiovascular manifestations

So far, I have not experienced any cardiovascular manifestations of concern. But I am aware they are possible. Along with the ramifications of inheritance, the geneticist recommended annual echocardiograms to screen for aortic root dilation and other valvular dysfunction.

## Discussion

The classic presentation of MFS includes spontaneous pneumothorax, which is why this was originally the working diagnosis. However, LDS and vascular EDS (vEDS) also present with spontaneous pneumothorax. Even more interesting, large joint hypermobility and skin hyperextensibility are not common in vEDS [4]. In fact, spontaneous pneumothorax has been shown to be a common manifestation in vEDS patients, with some studies claiming up to 18%. In these studies, 81% of pneumothoraces preceded the diagnosis of vEDS. The diagnosis of vEDS was made on average seven years after arterial or intestinal complications [5]. For those with recurrent pneumothoraces, bullectomy, pleural rubbing, and chemical pleurodesis are indicated [6].

There are cases of patients with MFS and LDS who undergo evaluation for GERD, but these are secondary to a structural abnormality (such as a hiatal hernia which is more commonly seen in these disorders) [7,8]. GERD has been a well-characterized manifestation of hypermobility EDS (hEDS), upwards of 50% in some studies [9]. GERD is refractory to high doses of PPIs in combination with H2-blockers and acid-neutralizing medications (such as calcium carbonate). In these patients, endoscopy and surgery appear to be the best options for symptom management; however, more evidence is needed [10].

Chronic fatigue is well characterized in both cEDS and hEDS with common findings such as poor sleep quality, chronic pain, physical deconditioning, orthostatic intolerance, headaches/migraines, and anxiety and/or depression. The incidence, prevalence, and natural history are unknown. However, studies have hypothesized that poor postural control, nocturnal skeletal pain, and cardiovascular dysautonomia contribute to exercise intolerance/muscle weakness, unrefreshing sleep, and post-exertional malaise. These phenotypes summate in the symptom of chronic fatigue [11]. Chronic fatigue is defined by: (1) persistent and/or recurrent fatigue present for more than six months, (2) unexplained by other conditions, (3) not the result of ongoing exertion, (4) not substantially alleviated by rest, and (5) resulting in substantial reduction or impairment in the ability to engage in normal levels of activities. The fatigue from EDS may be indistinguishable from CFS. As such, it is unclear how many patients diagnosed with CFS have EDS. The general management should be to treat the underlying cause. This may include counseling on sleep management, graded exercise therapy, or cognitive behavioral therapy [12,13].

The cardiovascular manifestations of MFS are well known and usually present later than the skeletal, ophthalmologic, and pulmonary complications, enabling earlier diagnosis and screening to prevent cardiovascular catastrophes. Cardiovascular manifestations of LDS differ with widespread aortic and arterial aneurysms instead of affecting the root or ascending aorta. Furthermore, manifestations in LDS patients are more severe with aneurysmal dissection or rupture at a smaller diameter and younger age. However, many LDS patients present similar to MFS patients with dilatation of the aortic root. Although up to one-quarter of EDS patients show aneurysmal disease most commonly of the descending thoracic or abdominal aortas, patients can also present with dilatation of the aortic root. EDS cardiovascular disease is most common in vEDS and hEDS presenting as abdominal aortic aneurysms in up to 32% and mitral valve prolapse in up to 6% of EDS patients [4,14].

Relatively recent literature has been published regarding the psychosocial impact of EDS. Of these qualitative interviews, the most common themes are (1) limitations in diagnosis and treatment, (2) physical restrictions, and (3) restructuring leisure and social relationships. Almost every interviewee experienced a delay in diagnosis and frustration with being called a “hypochondriac” or “sent away with no answers.” Even with the lack of effective treatment options, the value of a diagnosis brings immeasurable relief and validation to patients who live believing their symptoms are contrived. Furthermore, every interviewee stated their physical limitations by pain and fatigue led to abnormal lives and required adaptions. Patients report having a purpose, whether this is a job or other activity, has helped them cope. Many interviewees specifically mentioned the pelvic and musculoskeletal pain associated with intimacy as stress on their mental health and relationships. This leads to embarrassment and fear of rejection from partners. Interviewees mentioned the inability to “keep up” with their friends and family whether physically or socially. For example, patients would be so fatigued from cleaning their home, they would cancel dinner plans with family and friends [15,16].

Although I have a genetic mutation indicative of cEDS, I do not meet the diagnostic criteria for a clinical diagnosis. The involved genes and best-known diagnostic criteria for the reviewed inherited connective tissue diseases are listed in Table 1. This table is in no way comprehensive of every connective tissue disease but displays how the diseases which fit my symptomatology are diagnosed. It should be noted that hEDS is the only disease that does not have a clear genetic cause, making the disease more comprehensive and difficult for the clinician to officially diagnose. All listed diseases are known to have an autosomal dominant pattern of inheritance.

**Table 1:** Genes and diagnostic criteria for inherited connective tissue diseases. GJH: generalized joint hypermobility; EDS: Ehlers-Danlos syndrome Genetic variants and diagnostic criteria are shown in the table. Note that all diseases except hEDS require confirmation of genetic variant plus listed diagnostic criteria for an official diagnosis.

Author	Disease name	Gene(s)	Diagnostic criteria
Malfait et al. [17] (2017)	Classic EDS (cEDS)	*COL5A1*, *COL5A2*, *COL1A1*	Presence of skin hyperextensibility and atrophic scarring
			Plus GJH and/or at least three of the following: (1) easy bruising, (2) soft, doughy skin, (3) skin fragility, (4) molluscoid pseudotumors, (5) subcutaneous spheroids, (6) hernia (or history thereof), (7) epicanthal folds, (8) complications of joint hypermobility, and (9) family history of a first-degree relative who meets the clinical criteria
Malfait et al. [17] (2017)	Hypermobile EDS (hEDS)	Unknown	Presence of GJH
			Plus two or more of the following: (1) systemic manifestations of a more generalized connective tissue disorder, (2) positive family history, with one or more first-degree relatives independently meeting the current diagnostic criteria for hEDS, and (3) musculoskeletal complications
			Plus all of the following: (1) absence of unusual skin fragility, (2) exclusion of other heritable and acquired connective tissue disorders, and (3) exclusion of alternative diagnoses that may also include joint hypermobility by means of hypotonia and/or connective tissue laxity
Malfait et al. [17] (2017)	Vascular EDS (vEDS)	*COL3A1*, *COL1A1*	Minimum criteria for further diagnostic studies include any one of the following: (1) family history of the disorder, (2) arterial rupture or dissection in individuals less than 40 years of age, (3) unexplained sigmoid colon rupture, and (4) spontaneous pneumothorax
Dietz [18] (2017)	Marfan syndrome (MFS)	*FBN1*	Presence of at least one of the following: (1) aortic root enlargement (Z-score ≥2.0), and (2) ectopia lentis
Loeys and Dietz [19] (2018)	Loeys-Dietz syndrome (LDS)	*SMAD2*, *SMAD3*, *TGFB2*, *TGFB3*, *TGFBR1*, *TGFBR2*	Presence of at least one of the following: (1) aortic root enlargement (defined as an aortic root Z-score ≥2.0) or type A dissection, and (2) compatible systemic features including characteristic craniofacial, skeletal, cutaneous, and/or vascular manifestations found in combination

Colombi et al. recognized the lack of a systemic approach to differentiation and devised a diagnostic flowchart to distinguish joint hypermobility syndrome/hEDS from other similar presentations such as other EDSs, MFS, LDS, arterial tortuosity syndrome (ATS), OI, and lateral meningocele syndrome [20]. A more comprehensive diagnostic flowchart is necessary to encompass the systemic nature of these diseases. The utility of these flowcharts is limited due to the high presentation variability and extensive symptom overlap between these syndromes. Additionally, our current knowledge cannot fit each disorder into its own schematic. Instead, medical professionals should be aware of the multitude of inherited connective tissue diseases and be aware of the management of the most morbid complications.

## Conclusions

EDS is one of the many systemic connective tissue disorders which have variable presentations and extensive symptom overlap, making the diagnosis challenging and requiring proper assessment to prevent delay in diagnosis. Although there is no effective treatment for the genetic disorder, early diagnosis can help patients manage their condition successfully. Spontaneous pneumothorax and aortic aneurysm are common in patients with EDS and require more thorough investigation to avoid catastrophic complications. Determining an accurate diagnosis is not essential in the management of these syndromes as the genetic abnormality may not indicate clinical symptoms, such as in the described case.

## References

[1] Miklovic T, Sieg VC (2021). Ehlers Danlos syndrome. https://www.ncbi.nlm.nih.gov/books/NBK549814/.

[2] Malfait F, Castori M, Francomano CA, Giunta C, Kosho T, Byers PH (2020). The Ehlers-Danlos syndromes. Nat Rev Dis Primers.

[3] Islam M, Chang C, Gershwin ME (2021). Ehlers-Danlos syndrome: immunologic contrasts and connective tissue comparisons. J Transl Autoimmun.

[4] Meester JA, Verstraeten A, Schepers D, Alaerts M, Van Laer L, Loeys BL (2017). Differences in manifestations of Marfan syndrome, Ehlers-Danlos syndrome, and Loeys-Dietz syndrome. Ann Cardiothorac Surg.

[5] Shalhub S, Neptune E, Sanchez DE, Dua A, Arellano N, McDonnell NB, Milewicz DM (2019). Spontaneous pneumothorax and hemothorax frequently precede the arterial and intestinal complications of vascular Ehlers-Danlos syndrome. Am J Med Genet A.

[6] Chohan K, Mittal N, McGillis L (2021). A review of respiratory manifestations and their management in Ehlers-Danlos syndromes and hypermobility spectrum disorders. Chron Respir Dis.

[7] Eliashar R, Sichel JY, Biron A, Dano I (1998). Multiple gastrointestinal complications in Marfan syndrome. Postgrad Med J.

[8] Berger JA, Huh DD, Lee T, Wadia RS, Bembea MM, Goswami DK (2021). Perioperative management and considerations in pediatric patients with connective tissue disorders undergoing cardiac surgery. Paediatr Anaesth.

[9] Castori M, Morlino S, Pascolini G, Blundo C, Grammatico P (2015). Gastrointestinal and nutritional issues in joint hypermobility syndrome/Ehlers-Danlos syndrome, hypermobility type. Am J Med Genet C Semin Med Genet.

[10] Levy HP (2004). Hypermobile Ehlers-Danlos syndrome. https://www.ncbi.nlm.nih.gov/books/NBK1279/.

[11] Castori M, Morlino S, Celletti C (2013). Re-writing the natural history of pain and related symptoms in the joint hypermobility syndrome/Ehlers-Danlos syndrome, hypermobility type. Am J Med Genet A.

[12] Malfait F, Wenstrup R, De Paepe A (2007). Classic Ehlers-Danlos syndrome. https://www.ncbi.nlm.nih.gov/books/NBK1244/.

[13] Hakim A, De Wandele I, O'Callaghan C, Pocinki A, Rowe P (2017). Chronic fatigue in Ehlers-Danlos syndrome-hypermobile type. Am J Med Genet C Semin Med Genet.

[14] Cury M, Zeidan F, Lobato AC (2013). Aortic disease in the young: genetic aneurysm syndromes, connective tissue disorders, and familial aortic aneurysms and dissections. Int J Vasc Med.

[15] Palomo-Toucedo IC, Leon-Larios F, Reina-Bueno M, Vázquez-Bautista MD, Munuera-Martínez PV, Domínguez-Maldonado G (2020). Psychosocial influence of Ehlers-Danlos syndrome in daily life of patients: a qualitative study. Int J Environ Res Public Health.

[16] Bennett SE, Walsh N, Moss T, Palmer S (2021). Understanding the psychosocial impact of joint hypermobility syndrome and Ehlers-Danlos syndrome hypermobility type: a qualitative interview study. Disabil Rehabil.

[17] Malfait F, Francomano C, Byers P (2017). The 2017 international classification of the Ehlers-Danlos syndromes. Am J Med Genet C Semin Med Genet.

[18] Dietz H (2017). Marfan syndrome. https://www.ncbi.nlm.nih.gov/books/NBK1335/.

[19] Loeys BL, Dietz HC (2018). Loeys-Dietz syndrome. https://www.ncbi.nlm.nih.gov/books/NBK1133/.

[20] Colombi M, Dordoni C, Chiarelli N, Ritelli M (2015). Differential diagnosis and diagnostic flow chart of joint hypermobility syndrome/ehlers-danlos syndrome hypermobility type compared to other heritable connective tissue disorders. Am J Med Genet C Semin Med Genet.

